# Congratulatory Address to Samuel Gaskell, Esq.

**Published:** 1850-01-01

**Authors:** 


### CONGRATULATORY ADDRESS TO SAMUEL GASKELL, ESQ., F.R.C.S.E.

AND ONE OF HER MAJESTY'S COMMISSIONERS IN LUNACY, ETC. ETC.

Dear Sir,—We, Members of the Association of Medical Officers of Hospitals for the Insane, feeling highly gratified at the selection made by the Lord Chancellor of you, the Superintendent of the Lancaster County Lunatic Asylum, and a Member of this Association, to fill the office of Commissioner in Lunacy, take the earliest opportunity of expressing our feelings and congratulations to you; and the assurance that, in our opinion, no one could have been appointed to that office more qualified to discharge its duties efficiently, or more calculated to give satisfaction to those of the medical profession with whom you will thereby be brought into contact.

We fervently hope that you may long enjoy good health to hold the same, and to assist us (in your now more elevated position) in the accomplishment of those objects—viz., "The Improvement of Lunatic Asylums, the Treatment of the Insane, and the Acquirement of a more extensive and more correct Knowledge of Insanity"—for which the Association was founded.

And we remain, dear Sir,

Sincerely and faithfully yours,

J. S. ALDERSON, M.R.C.S., Superintendent of Nottingham Lunatic Asylum.
W. C. BEGLEY, M.D., Resident Physician to Hanwell Lunatic Asylum.
ROBERT BOYD, M.D., Res. Phy. and Sup. of Somerset County Lun. Asylum.
JOHN BROADHURST, M.R.C.S., Med. Sup. of Lancaster Co. Lun. Asylum.
J. C. BUCKNILL, M.D., Res. Phy. and Sup. of Devon Co. Lun. Asylum.
W. M. BUSH, M.D., Sandywell Park, Gloucestershire.
JOHN CONOLLY, M.D., Vis. Phy. to the Hanwell Lun. Asylum.

C. C. CORSELLIS, M.D., Res. Phy. and Sup. of the Asylum for West Riding of York.
E. D. DE VITRE, M.D., Vis. Phy. to Lancaster Co. Lun. Asylum.
JOHN HITCHMAN, M.D., Res. Phy. to Hanwell Lun. Asylum.
JOHN HOLLAND, M.R.C.S., Res. Med. Officer and Sup. of Surrey Co. Lun. Asylum.
JAMES E. HUXLEY, M.D., Res. Phy. and Sup. of Kent Lun. Asylum.
GEO. T. JONES, M.R.C.S., Sup. of North Wales Lun. Asylum.
J. KIRKMAN, M.D., Res. Phy. and Sup. of Suffolk Lun. Asylum.
WM. LEY, M.R.C.S., Med. Sup. of Lunatic Asylum for Cos. of Oxford and Berks.
RICHARD MALLAM, M.R.C.S., Proprietor of the Hook Norton Licensed House.
SIR A. MORISON, M.D., Vis. Phy. to Bethlem Hosp. and Surrey Co. Lun. Asylum.
P. R. NESBITT, M.D., Res. Phy. and Sup. of Northampton Lun. Asylum.
RICH. OLIVER, M.D., Med. Sup. of Lun. Asy. for Cos. of Salop and Montgomery.
H. F. PROSSER, M.R.C.S., Med. Sup. of Leicester Co. Lun. Asylum.
ROBERT STEWART, M.D., Res. Phy. and Sup. of Belfast District Lun. Asylum.
A. J. SUTHERLAND, M.D., F.R.S., Phy. to St. Luke's Hosp. London.
SAMUEL S. THOMPSON, M.D., Vis. Phy. to Belfast Dis. Lun. Asylum.
JOHN THURNAM, M.D., Med. Sup. of Wilts. Co. Lun. Asylum.
D. T. TYERMAN, M.R.C.S., Med. Sup. of Co. Lun. Asylum, Cornwall.
F. D. WALSH, M.R.C.S., Med. Sup. of Lincoln Lun. Asylum.
R. LOYD WILLIAMS, M.D., Vis. Phy. to North Wales Lun. Asylum.
J. C. WILLIAMS, M.D., Vis. Phy. to Nottingham Lun. Asylum.
F. T WINTLE, M.D., Res. Phy. to Warneford Hosp. for Insane.
JAMES WILKES, M.R.C.S., Med. Sup. of Stafford Co. Lun. Asylum.
SAMUEL HITCH, } Joint Secretaries to the Association of Medical Officers of Hos-
W. W. WILLIAMS, } pitals for the Insane.

This Address was inadvertently omitted in our last Number.

